# The role of initiator on the dispersibility of polystyrene microgels in non-aqueous solvents

**DOI:** 10.1007/s00396-017-4023-y

**Published:** 2017-02-07

**Authors:** Jessica A. Bonham, Franceska Waggett, Malcolm A. Faers, Jeroen S. van Duijneveldt

**Affiliations:** 1 School of Chemistry, University of Bristol, Cantock’s Close, Bristol, BS8 1TS UK; 2 Bayer AG, Crop Science Division, Alfred-Nobel-Str. 50, D-40789 Monheim, Germany

**Keywords:** Colloid, Polymer, Microgel, Initiator, Swelling

## Abstract

Non-aqueous microgel particles are commonly synthesised in water, dried, and then redispersed in non-aqueous solvents. An important factor to consider when synthesising such particles is the initiator, which can determine how well the particles disperse in solvents. Polystyrene microgel particles were made with three different initiators. When a neutral, oil soluble initiator (azobisisobutyronitrile) was used the particles dispersed in toluene as well as cyclohexane and decalin. In contrast, anionic, water-soluble initiators (potassium persulfate or azobis(4-cyanovaleric acid)) created particles that only redispersed in toluene and not the other two solvents. Of the three considered, toluene is the best solvent for polystyrene and also has the highest polarizability, making it most effective at redispersing particles with polar or ionisable functional groups. Zeta potential and conductivity measurements, however, did not detect a direct relationship between particle charging and redispersibility. Oil soluble initiators result in “inside out” polymerisation where the initiator groups are buried inside the growing particle, whereas water-soluble initiators result in “outside in” polymerisation, with the polar initiator groups residing on the particle surface. By tailoring the ratio between water and oil soluble initiators, it may be possible to synthesise microgel particles with uniform or designed charge profiles from the core to the surface.

**Electronic supplementary material:**

The online version of this article (doi:10.1007/s00396-017-4023-y) contains supplementary material, which is available to authorized users.

## Introduction

Non-aqueous polymer microgels are often made in poor solvents, notably water, where they are un-swollen, and then transferred into good solvents where the particles swell [1]. The polymer–solvent interaction parameter, *χ* is a key factor that controls whether or not particles will redisperse in solvent. The choice of both the monomer and solvent governs the *χ* parameter, as does the temperature. To what extent particles do swell in good solvents is determined by the molar mass between cross-links, as shown by the Flory-Rehner theory [2]. Here, we demonstrate that, in addition to these properties of the bulk gel, the nature of the particle surface also needs to be considered, and the initiator used during particle synthesis can influence whether the microgel particles will disperse in a given solvent [3].

There are two main ways by which the initiator can influence the properties of a microgel particle. Firstly, the initiator is responsible for functional groups on the particles. During polymerisation, free radicals from the initiator become incorporated into the end groups of the polymer chains and hence any functional or polar groups on the initiators will lead to functionalized or polar particles [4–6]. Nair [5] made microgel particles using two different initiators, 4,4 ^′^-azobis(4-cyanovaleric acid) (ACVA) and potassium persulfate (KPS) and found that particles made with ACVA could be redispersed in low polarity solvents, whereas KPS particles could only be redispersed in higher polarity solvents. They attributed this to the weakly acidic surfaces and low surface charge densities of ACVA particles compared to the strongly acidic surfaces and high surface charge densities of the KPS particles [5].

The solubility of the produced initiator radicals also affects the particle properties as it determines where the polymerisation occurs and where on the particle the functional groups will reside [4, 7]. If the particle surface is covered in functional polar groups from the initiator molecules, then the overall particle dispersibility will be affected whereas, if the initiator molecules are hidden within the particles, then the particle dispersibility will be less dependent on the initiator functionality. Mori and Kawaguchi [7] made magnetic polymer particles using a miniemulsion polymerisation and found that both the size of the particles and the location of the magnetite depended on the solubility of initiator used [7]. The polymerisation kinetics, droplet nucleation mechanism [4] and interactions with surfactants [6] are also affected by the type of initiator.

Whilst it has been shown that the initiator affects the type and location of functional groups on microgel particles in non-aqueous solvents, there have been no studies in the literature on the combination of these two aspects and how this would influence the redispersion of microgel particles. Furthermore, in non-aqueous solvents, the role of polar or ionic functional groups are of particular interest as charges are difficult to stabilize due to the low dielectric constants [8, 9]. Understanding such systems would enable more accurate predictions to be made on the dispersion of particles, benefitting both industrial and fundamental applications of microgel particles in non-aqueous solvents.

In this work, polystyrene microgel particles have been synthesised using three different initiators and swollen in toluene, decalin and cyclohexane. The effect of the initiator type on the particle redispersion has been studied using dynamic light scattering. Conductivity and zeta potential measurements of the particles in various solvents have also been carried out.

## Materials and methods

### Materials

Styrene (S), divinylbenzene (55 %) (DVB), potassium persulfate (KPS), azobis(4-cyanovaleric acid) (ACVA), tetrabutylammonium bromide (TBAB) and sodium dodecylbenzenesulfonate (SDBS) were purchased from Sigma-Aldrich.

Azobisisobutyronitrile (AIBN) was purchased from Acros Organics, dodecyltrimethyl ammonium bromide (DTAB) was purchased from Molekula and diisopropylbenzene (DIB) was purchased from T.C.I.. Toluene was purchased from Sigma-Aldrich and cyclohexane and decalin were purchased from BDH Chemicals Ltd.

Prior to the reaction, the styrene was run through a column to remove any inhibitor. Styrene, AIBN, KPS, ACVA and DVB were kept at 5 ^∘^C before use and the solvents were dried using molecular sieves before use.

### Microgel synthesis

Table 1 summarises the details of the microgel particles including the initiator and cross-linker used, the reaction temperature and time. The cross-link density is quoted as the molar ratio of monomer:cross-linker. M1, M5 and M6 microgel particles were made via a microemulsion polymerisation [10], M2 microgel particles were made via an emulsion polymerisation [11] and M4 microgels were made via a surfactant free emulsion polymerisation [1, 12]. In the following sections, the experimental set-up for each type of reaction is given. For all microgel batches, an aliquot of product was set aside before drying the particles, in order to obtain DLS sizes of the particles in water. For a more detailed description of the different microgel synthesis routes, see Bonham et al. [2].

**Table 1:** Experimental conditions

Sample	Initiator	Cross linker	Cross link density	Temperature/ ^∘^C	Time/hours	Polymerisation type	Surfactant
M1	AIBN	DVB	1/80	65	72	MEP	DTAB
M2	KPS	DVB	1/80	70	24	EP	SDBS
M4	ACVA	DIB	1/80	70	21	SFEP	N/A
M5	AIBN	DIB	1/80	65	24	MEP	DTAB
M6	AIBN	DIB	1/44	65	24	MEP	DTAB

*MEP* microemulsion polymerisation, *EP* emulsion polymerisation, *SFEP* surfactant-free emulsion polymerisation

#### Micro-emulsion polymerisation

The synthetic method given below is for M6 particles where a total reaction mixture of 250 g was made; a similar method was employed for M1 and M5 microgel particles. Then, 9.1 wt% styrene was mixed with 0.32 wt% DIB and 0.045 wt% AIBN and added to a 3-necked round bottomed flask connected to a reflux condenser. Furthermore, 85.4 wt% water and 5.1 wt% DTAB were added and left to stir at 300 rpm under an atmosphere of argon for 1 h. The reaction was heated for 24 h at 338 K. During this time, a milky white solution formed. The reaction was then left to cool and washed through glass wool to remove any partially polymerised residues. The particles were washed with hot methanol and re-dispersed in tetrahydrofuran before being washed with cold methanol a further three times. A chloride analysis was used to confirm that the surfactant had been fully removed after this process. The resulting white powder was then dried in a vacuum oven for 42 h at 323 K and a product yield of 93 % was obtained.

#### Emulsion polymerisation

M2 microgel particles were made via an emulsion polymerisation according to ref. [11]. 90.22 wt% water was mixed with 0.14 wt% SDBS and stirred in a 3-necked round bottomed flask, connected to a reflux condensor at 500 rpm under an atmosphere of argon for 10 min. Then, 3.22 wt% styrene and 0.05 wt% DVB were then added and stirred for a further 15 min. Furthermore, 0.12 wt% KPS initiator, dissolved in 3.75 wt% water and washed with another 2.5 wt% water, was then added and the reaction was heated at 338 K for 24 h.

After cooling to room temperature, the product was run through glass wool to remove any partially polymerised residues and then dialysed for 2 weeks, until the conductance was stable. The water was removed using a rotary evaporator and the remaining white powder was dried in a vacuum oven overnight. A product yield of 87 % was achieved.

#### Surfactant-free emulsion polymerisation

M4 microgel particles were made via a surfactant free emulsion polymerisation (SFEP) according to ref. [1] and [12]. The experimental setup for SFEP was the same as for the previous experiments.

Then, 87.86 wt% water, adjusted to pH 9 using NaOH, was added to the reaction flask and heated to 343 K under an argon atmosphere. Furthermore, 9.55 wt% styrene and 0.19 wt% DIB were then added to the flask. ACVA (0.08 wt%) was dissolved in 2.32 wt% water, which was adjusted to pH 11 using NaOH. The reaction was left to stir for 21 h at 343 K.

After the reaction cooled to room temperature, the solution was passed through glass wool and centrifuged with water 7 times at 10,000 *g* for 30 mins each time. The sample was then dried in a rotary evaporator and then in a vacuum oven overnight after which a product yield of 90 % was achieved.

#### Re-dispersing particles in non-aqueous solvents

The dried microgel particles were re-dispersed in various organic solvents. 0.015 g of microgels were dissolved in 3 ml solvent and placed in a small vial. The vials were homogenised by tumbling overnight. If a dispersion was formed, the particles remained suspended in the solvent even when left to stand. If the particles were unstable in the solvent, they sedimented to the bottom of the vial when left to stand. For samples that did not initially disperse after tumbling, additional dispersion techniques were employed, for example heating the samples, placing them in a sonic bath (IND 500D, Ultrawave) and using a pulsed tip sonicator (QSonica Q125, 125 W) for 5 mins. None of these techniques improved the dispersion of the particles. For cyclohexane, the particles only formed stable dispersions when the temperature was above 303 K, in order to achieve this the vials were placed in an incubator for at least 2 h to equilibrate.

### Particle characterisation

Dynamic light scattering (DLS) was carried out to determine the hydrodynamic diameter, *d*_H_ of the microgel particles using a Malvern Autosizer 4800; the temperature was controlled using an external water bath. The samples were dissolved in fresh solvent at concentrations of ca. 5 mg ml ^−1^ and the solutions were filtered with a 5 μm Millipore filter to remove any dust particles. Readings were taken multiple times to ensure reproducible results.

The swelling of a microgel particle is described by the volume swelling ratio, *q*, which can be expressed empirically as a ratio of hydrodynamic diameters of swollen (*d*_H_) and collapsed (*d*_0_) particles, see Eq. 1. In general (especially for aqueous microgels), particles may still contain solvent in the collapsed state. However, for a system of hydrophobic particles, such as PS microgels, it is assumed that particles in water are fully collapsed with no solvent present and that particles in non-aqueous solvents are swollen. With this in mind, *q* will refer to the swelling ratio as defined below throughout this work.

1 $$ q = \left( \frac{d_{\text{H}}}{d_{0}} \right)^{3}  $$

The conductivity and electrophoretic mobility of the particles was determined using a Malvern Zetasizer Nano ZS with a laser wavelength of 633 nm at 298 K. For samples in water, a disposable cell was used and for samples in non-aqueous solvents, a non-aqueous dip cell was used. To prepare microgel solutions in water, clean particles, where the surfactant had been removed by dialysis or a methanol work-up, were redispersed in water at a concentration of ca. 0.1 mg ml ^−1^. Without added surfactant, the particles were no longer soluble in water and hence a tip sonicator (QSonica Q125) was used in pulse mode at 80 % max power for 25 min to disperse the particles. When needed, NaCl (aq) was added to the suspension after the particles were dispersed. The solutions were filtered with a 5 μm Millipore filter to remove any dust particles and readings were taken multiple times to ensure reproducible results.

The conductivities of the microgel particles in non-aqueous solvents were measured using a cylindrical concentric stainless steel conductivity probe (Model 627, Scientifica) at 298 K.

## Results and discussion

Five different microgel batches are synthesised with various reaction conditions, see Table 1 and *d*_H_, measured using DLS, of the microgel particles in water, toluene, decalin and cyclohexane is shown in Table 2. The relative swelling of each particle and the effect of solvency, cross-linker and cross-link density will not be discussed in detail here. Toluene is a better solvent for polystyrene than cyclohexane or decalin and it is expected that the particles will be largest in toluene. This is the case for both M1 and M6 particles; however, for M5 particles, the swelling ratio in cyclohexane is very similar to that of toluene. Furthermore, these particles have large error bars which suggest that they are on the verge of aggregation.

**Table 2:** Dynamic light scattering and zeta potential results for particles in various solvents

Sample	Initiator	*d*_0_ in	*d*_H_ in	*d*_H_ in	*d*_H_ in cyclo-	*ζ* in	*ζ* in	*v*_p_^b^
		water/nm	toluene/nm	decalin/nm	hexane^a^/nm	1 μM NaCl(aq)/mV	deionised water/mV	
M1	AIBN	122 ±2	182 ± 1	148 ± 3	154 ± 2	−26 ± 2	−22 ± 8	35,000
M2	KPS	71 ± 1	114 ± 3	−	−	−17 ± 1	−34 ± 2	31,000
M4	ACVA	463 ± 9	840 ± 5	−	−	−15 ± 1	−28 ± 1	1.9×10^6^
M5	AIBN	53 ± 1	84 ± 1	75 ± 1	82 ± 2	−18 ± 1	−24 ± 1	2,900
M6	AIBN	52 ± 1	79 ± 1	62 ± 1	69 ± 7	−25 ± 1	−29 ± 1	2,700

^a^ Experiment ran at 303 K

^b^ *v*_p_= possible number of charges per particle, determined using the particle size and initiator concentration

### Effect of type of location of initiator

**Fig. 1:**
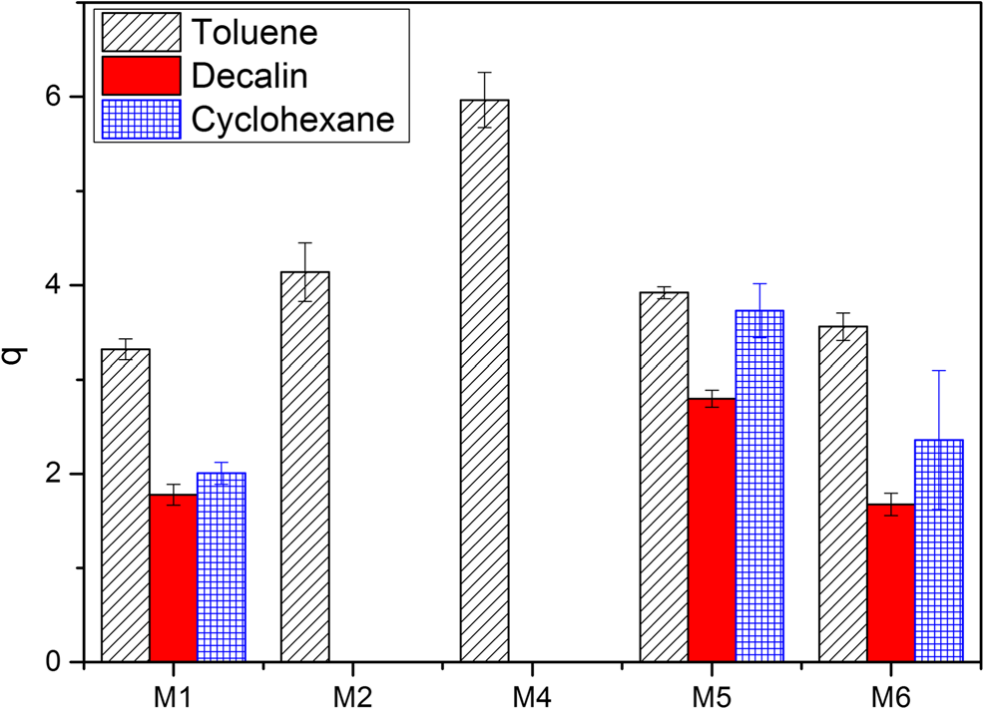
The volume swelling ratio (*q*) of the microgel particles in toluene, decalin and cyclohexane

The type of initiator used greatly affects the redispersion of the particles, as only when an oil soluble AIBN initiator is used, do the particles disperse in all three solvents. It could be argued that in cyclohexane, the particles are only just soluble, as there is evidence that they are beginning to aggregate. When a water-soluble initiator is used, KPS for M2 and ACVA for M4, the particles do not disperse in decalin or cyclohexane, see Fig. 1.

**Fig. 2:**
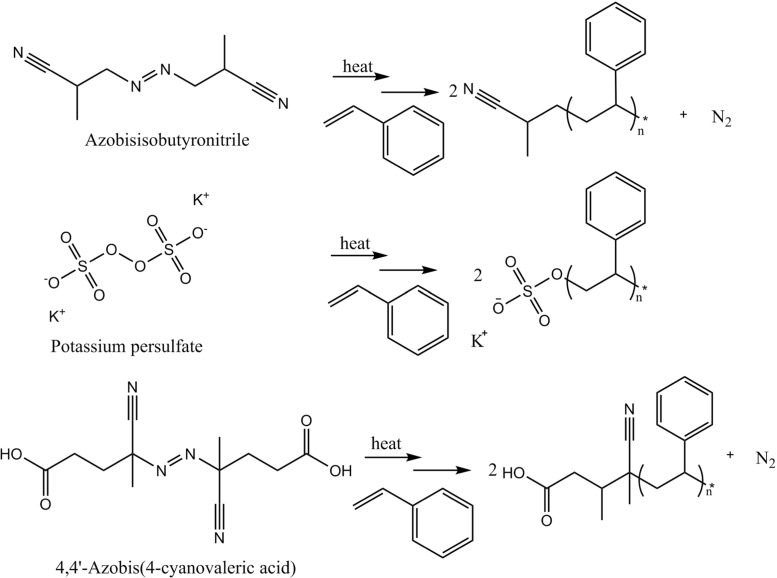
Schematic of the three different initiators used and resulting polymer structures

**Fig. 3:**
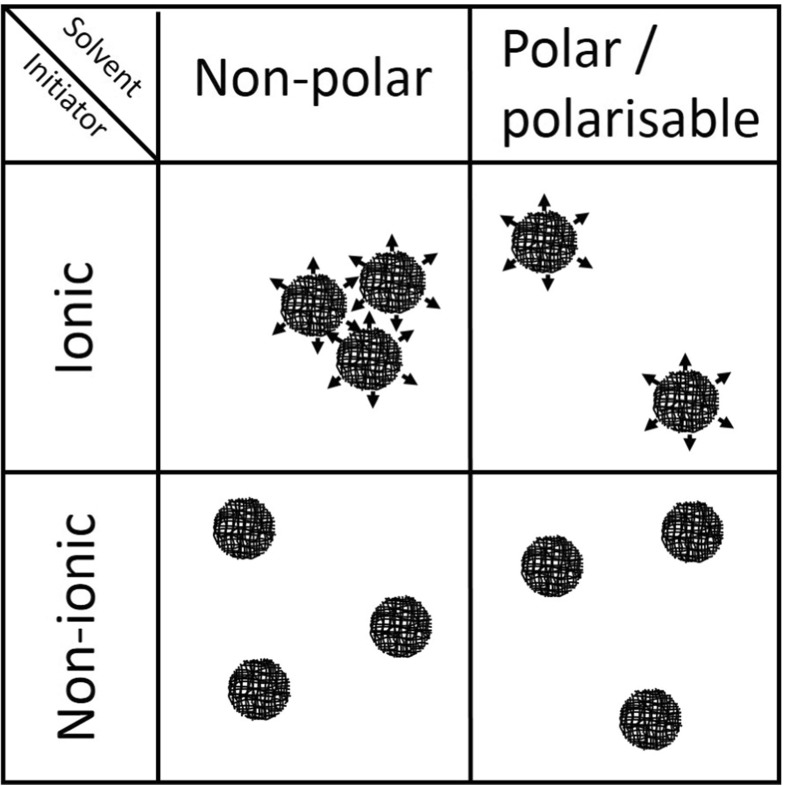
The mechanism of ionic and non-ionic particle dispersion in non-polar and polar or polarisable solvents. Ionisable functional groups are are illustrated as *arrows*

During synthesis, the functional groups on the initiator become covalently attached to the particles, which affects the dispersion properties of the resulting particles [3, 5, 6]. KPS produces anionic sulfate groups during propagation whereas ACVA is known to give clean carboxylic acid groups which can easily be ionised to give carboxylate anions [5]. AIBN, on the other hand, produces polar yet neutral cyano groups, see Fig. 2. Therefore, particles made with a KPS or ACVA will be potentially ionic and more polar than particles made with an AIBN initiator. The presence of polar functional groups is represented in Fig. 3 by arrows on the particle surface.

Of the three solvents, toluene is the only solvent to disperse these more polar KPS or ACVA particles. Toluene is a better solvent for polystyrene than decalin or cyclohexane, with the lowest *χ* parameter. The more favourable polymer–solvent interactions in toluene could be enough to overcome the unfavourable electrostatic interactions from the ionic initiator. In decalin or cyclohexane, the polymer–solvent interactions are less favourable and may not be sufficient to overcome these electrostatics. Furthermore, whilst the dielectric constant of toluene (2.38) is only marginally higher than that of decalin (2.15) or cyclohexane (2.02), toluene is an aromatic solvent and is consequently polarizable. The solubility of polar molecules, for example water, is higher in toluene (0.06 wt% [13]), a known hygroscopic solvent [14] than in decalin (0.01 wt% [13]) or cyclohexane (0.02 wt% [13]). Thus, toluene will be better at dispersing polar KPS and ACVA microgel particles than decalin and cyclohexane due to its aromaticity. Particles made with an AIBN initiator are less polar and therefore can still be stabilised in decalin and cyclohexane. These results are in agreement with the literature [5].

Figure 2 shows the proposed mechanism of both ionic and non-ionic particle dispersion in non-polar and polar or polarisable solvents. Non-ionic particles can be dispersed in polar and non-polar solvents as there are no dipole interactions. Particles made with an ionic initiator, however, are only stable in polar or polarisable solvents which can stabilise the polar surface groups.

The solubility of the initiator also affects the properties of the resulting particles as it governs where the initiator radicals are generated [4, 7]. M2 and M4 particles, made via emulsion polymerisation and surfactant-free emulsion polymerisation, respectively, have water-soluble initiators which decompose in the water phase before entering the monomer micelles. During micro-emulsion polymerisation, which was used for the synthesis of the other microgel particles, the oil-soluble initiators decompose inside the monomer droplets and thus produce radicals in the oil phase [2, 7]. It is expected that water-soluble KPS and ACVA initiators will produce “outside-in” particles, where polymerisation starts on the periphery of the particle and continues inwards, leaving ionic surface groups on the particle surface [15]. Oil-soluble, AIBN initiators should produce “inside-out” particles, where radicals are formed in the droplet and polymerisation extends outwards; leaving neutral particles [7].

### Conductivity and electrophoretic mobility in water

To quantify these particle charges, the conductivity, *κ* and the electrophoretic mobility, *μ*_*e*_ of the microgel particles in both deionised water and a 1 μM NaCl(aq) solution was determined. To see the charges on the particles in the absence of surfactant, the clean particles, i.e. after the surfactant was removed with dialysis or a methanol work up, were dispersed in water, see “Particle characterisation” section.

**Fig. 4:**
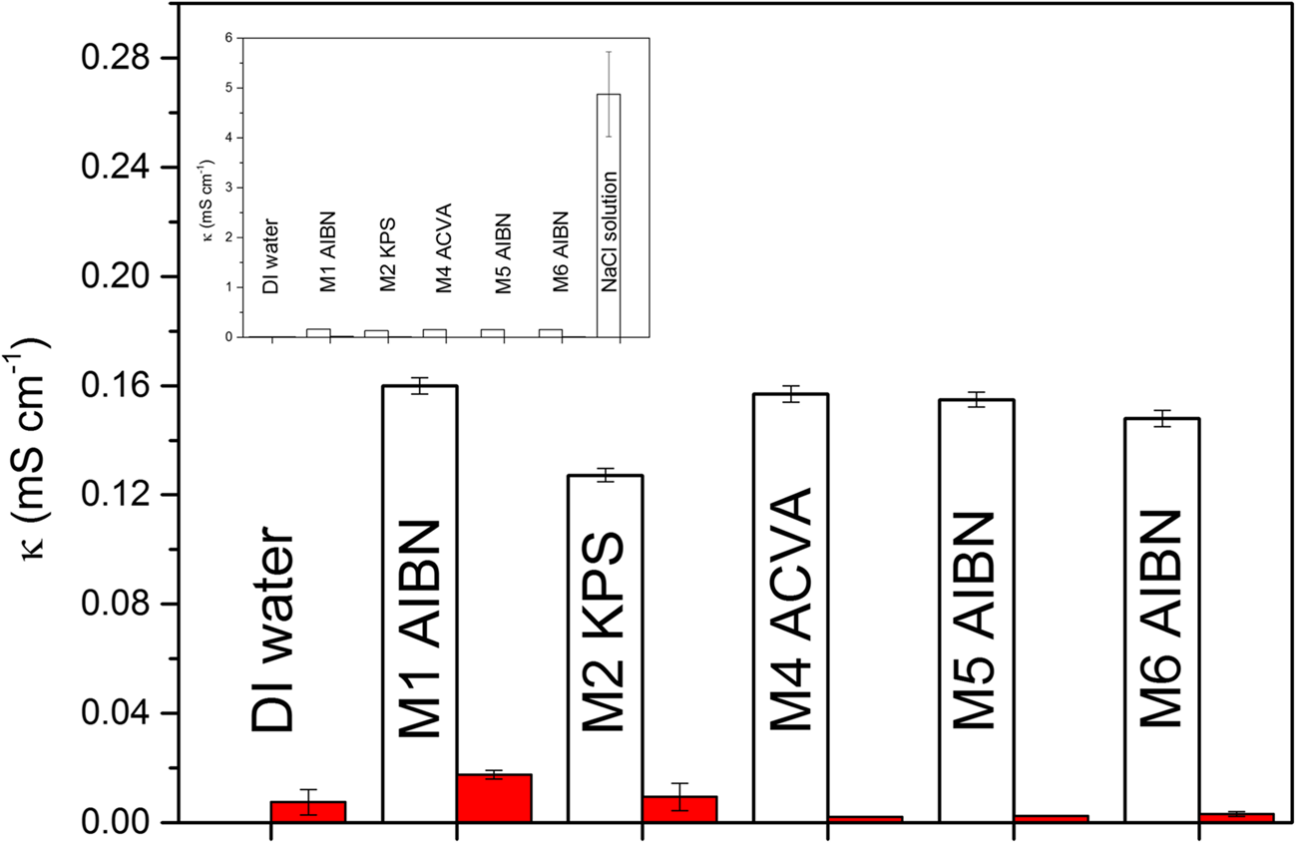
The conductivity of the microgel suspensions in a 1 μM aqueous solution of NaCl (*white open bars*). The conductivity of the particles in deionised water is also shown (*red filled bars*); the inset shows the conductivity of the NaCl solution. The concentrations are ca. 0.1 mg ml ^−1^

In deionised water, the conductivities of the microgel suspensions are very low and only M1 particles have a conductivity that is significantly higher than pure deionised water, see Fig. 4. In a 1 μM NaCl(aq) solution, the conductivities of all the microgel particles are significantly higher than pure deionised water yet considerably lower than a pure 1 μM NaCl(aq) solution. Chloride ions from NaCl are known to preferentially absorb onto polystyrene latex particles [16] which could explain why the conductivity of the microgel solutions is lower than the NaCl solution. However, the conductivities are so much lower than the NaCl solution, that it is possible that the sodium ions are also adsorbing to the particles. In both deionised water and a 1 μM NaCl(aq) solution, there is no trend in the conductivity value with respect to the type of initiator used during particle synthesis, as all the particles have very similar conductivities, see Fig. 4.

The zeta potential, *ζ*, was determined from the electrophoretic mobility, *μ*_*e*_, using the Henry Equation:

2 $$ \mu_{e} =\frac{2\varepsilon\zeta f(\kappa\alpha)}{3\eta} \text{,}  $$

where *ε* is the dielectric constant, *f*(*κ* *α*) is Henry’s function and *η* is the solvent viscosity. For an aqueous sample, the Smoluchowski approximation can be used where *f*(*κ* *α*) = 1.5. In non-aqueous samples, the Hückel approximation can be applied where *f*(*κ* *α*)=1 [17].

The zeta potential measurements were taken in both pure deionised water and a 1 μM NaCl(aq) solution; all the microgel particles have a negative *ζ* that is similar for the different initiators, see Table 2. In contrast to the conductivity measurements, *ζ* for all the particles except M1, is higher in pure deionised water than in the NaCl solution. One reason for this could be that hydroxyl ions could be associating with particles in the same manner as the chloride ions. The fact that the zeta potentials are typically lower (less negative) when adding 1 μM NaCl seems to contradict the finding that chloride ions adsorb specifically [16], indeed perhaps our particles adsorb sodium ions preferentially. At such low salt concentrations, the effect of charge screening on the zeta potential should not be significant. The origin of the negative *ζ* in M2 and M4 particles can be explained by the incorporation of anionic initiator groups into the particle. For AIBN particles, however, where the initiator groups are neutral, the negative zeta potentials are unexplained, particularly in pure deionised water, where there are no chloride ions to adsorb onto the particles.

It has been shown that polystyrene latex particles made with a hydrogen peroxide initiator contain carboxyl groups on the particle surface only. Although the exact mechanism for the formation of such groups is unknown, one possible explanation is that in the presence of oxygen, a peroxide is formed with styrene, which subsequently decomposes to produce an alcohol and then a carboxyl under oxidising conditions [15]. Such unwanted side reactions with oxygen are the prime reason that the polymerisation reactions in this work were carried out under an argon atmosphere. Nonetheless, it is possible that a similar type of reaction is responsible for the negative *ζ* seen for the AIBN initiated particles.

In water, it can be concluded that all the initiators form anionic particles, irrespective of the location or type of the initiator groups. There is no evidence that anionic initiators produce more highly charged particles than neutral initiators which is unexpected. For AIBN initiated particles, the zeta potential and conductivity measurements, resulting from the polar cyano groups, are similar to KPS or ACVA measurements even though the initiator groups should be on the inside of the particles. This is also unexpected as it was thought that groups on the inside of a particle would not affect the zeta potential or conductivity, particularly in a bad solvent such as water, where the particles are collapsed.

### Conductivity and electrophoretic mobility in non-aqueous solvents

In non-aqueous solvents, however, the role of charges is often ignored due to the low dielectric constants; there is a higher energy for charge dissociation in non-aqueous solvents compared to water [8]. Nonetheless, all colloidal particles are charged to some extent [18] and charges in non-aqueous solvents have long been known, with reports of charges in benzene as early 1952 [19]. Non-aqueous charges are an important concern in a number of industries and applications, for example in electrophoretic displays and in printers and photocopiers [9]. Dissociation of a small number of charges in non-aqueous solvents can lead to large Debye lengths and create long ranged repulsive interactions in comparison to the particle size, even when screened [18].

Surfactants are commonly used to stabilise charges in non-aqueous solvents [9] and there is little evidence for stabilised charged particles, such as microgels, in non-aqueous solvents in the absence of surfactant. Therefore, it is expected that the number of ionised initiator groups and hence the charge density of these microgel particles in non-aqueous solvents will be minimal.

**Fig. 5:**
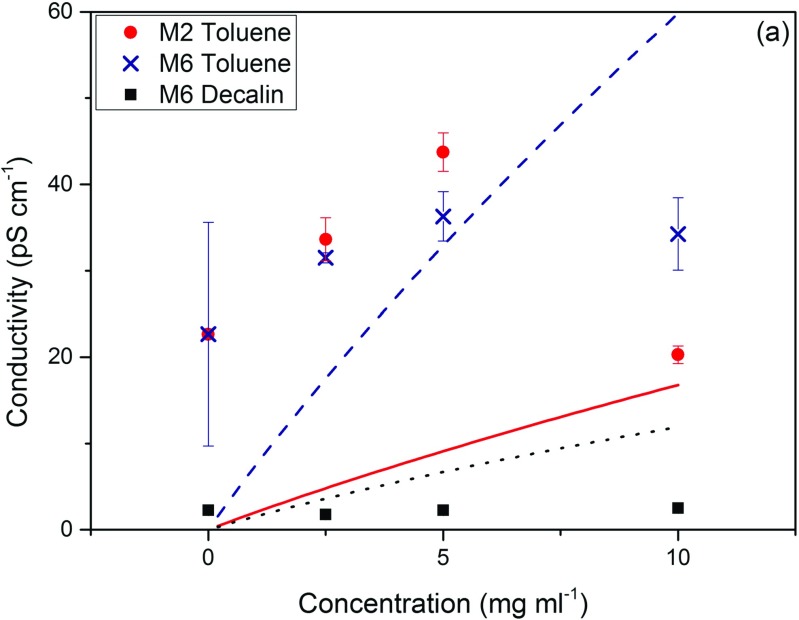
The conductivity in pS cm^−1^ as a function of concentration, of M2 particles in toluene (*red circles*), M6 particles in toluene (*navy crosses*) and M6 particles in decalin (*black squares*). *Lines* are theoretical values calculated using the Debye Hückel Onsager theory assuming a valency of 1, see Supplementary information. *Solid red line* = M2, *navy dashed line* = M6 in toluene and *black dotted line* = M6 in decalin

Measuring charges in non-aqueous solvents is notoriously difficult and it was not possible to measure the electrophoretic mobility of the microgel particles in non-aqueous solvents directly, using phase analysis light scattering, as the charges were too inconsistent and small to provide reliable readings. Therefore, the conductivities of M2 and M6 microgel suspensions in toluene and / or decalin were measured with a highly sensitive conductivity probe as a function of concentration; the conductivity of the pure solvent was also measured, see Fig. 5. The conductivities were difficult to accurately measure and often produced noisy results, particularly for larger M4 and M1 particles, where the number of particles is reduced due to the increased particle size. These results have consequently been omitted.

According to the literature, the conductivity of pure toluene is <10 pS/m at 293 K [20, 21]. The solvent conductivity measured here is 23 pS/m yet the error bars are also large. This suggests that there are some impurities in the solvent that are causing this conductivity. Adding particles does not necessarily equate to an increase in conductivity and in a number of cases, decreases the conductivity. The conductivities show no clear trend with concentration, particularly for M6 particles. For M2 particles in toluene, the conductivity does increase with concentration, until 10 mg ml ^−1^ where the conductivity decreases again. For M6 particles, the conductivities in decalin are clearly lower than in toluene however the very low conductivities in decalin are much harder to accurately measure than in toluene and thus no other particles in decalin could be measured.

The number of possible charges per particle, *v*_p_, determined using the particle size and initiator concentration, varies from 2700 for M6 particles to almost 2 million for M4 particles, see Table 2. In water, a significant proportion of these groups could ionise, and thus high conductivities are reported. In non-aqueous solvents, however, it is unfavourable to form ions and thus it is likely that only a handful of these groups ionise.

The conductivities in Fig. 5 have been compared to the Debye Hückel Onsager theory, where it is assumed that there is only one ionised group per particle, see the Supplementary information. At best the charges on the microgel particles are of order one charge per particle, or even less for M6 particles in decalin. Whilst there are significant error bars on the data, there is no clear trend of conductivity increasing with particle concentration, suggesting that a limiting concentration of ionised surface groups is reached already at these modest particle concentrations.

## Conclusions

It has been shown that the initiator used during particle synthesis has a direct impact on how well the particles will redisperse in various organic solvents. Polystyrene microgel particles made with a KPS or ACVA initiator can only be redispersed in an aromatic, polarisable solvent, whereas AIBN initiated particles are redispersible in all the solvents used. It is thought that the more polar groups on the KPS and ACVA initiators prevent particle redispersion in decalin and cyclohexane whilst toluene is able to overcome the polar interactions due to more favourable polymer–solvent interactions. Furthermore, toluene is polarisable and known to solubilise polar molecules better than decalin or cyclohexane.

It is also considered that during emulsion polymerisation with water-soluble initiators, the reaction initiates on the periphery of the particles and continues inwards, producing particles with the initiator groups on the particle surface. During micro-emulsion polymerisation with oil-soluble initiators, however, the reaction begins on the inside of the particle and continues outwards, with the initiator molecules hidden on the inside of the particles. Therefore, both the initiator solubility and polarisability are important parameters to consider when designing and synthesising non-aqueous microgels or latex particles. Furthermore, by tailoring the ratio between water- and oil-soluble initiators, it could be possible to synthesise microgel particles with uniform or designed charge profiles from the core to the surface.

Whilst it is known that initiators introduce charges into microgel particles, zeta potential and conductivity measurements did not demonstrate a difference between the different types of particles and the initiators used and were unable to prove a direct relationship between redispersibility and particle charging. Such measurements are notoriously difficult in non-aqueous solvents, hence additional experiments to study this relationship and quantify the location and charge of these particles are required.

Potentiometric and conductometric titrations can be used to accurately detect any potential charges on the particles made with KPS or ACVA initiators and therefore calculate the particle charge densities [5]. For AIBN-initiated particles, however, such titrations would still not show the presence of the neutral cyano groups on the particles.

## Electronic supplementary material

Below is the link to the electronic supplementary material.

(PDF 245 KB)

(TEX 9.29 KB)

(AUX 821 bytes)

(LOG 16.5 KB)

(GZ 16.8 KB)
