# Supplementary material for: The role of initiator on the dispersibility of polystyrene microgels in non-aqueous solvents
Source: Colloid Polym Sci. 2017 Feb 7;295(3):479–86. doi: 10.1007/s00396-017-4023-y (PMC5321690; doi:10.1007/s00396-017-4023-y)
Supplement: Supplementary file 1 — (PDF 245 KB) [file 396_2017_4023_MOESM1_ESM.pdf]

The Role of Initiator on the Dispersibility of Poly(styrene) Microgels in Non-aqueous Solvents

Jesscica A. Bonham*[a], Franceska Waggett[a], Malcolm A. Faers[b] and Jeroen S. van Duijneveldt[a]

[a]School of Chemistry, University of Bristol, Cantock's Close, Bristol, BS8 1TS, UK
[b]Crop Science Division, Bayer CropScience Aktiengesellschaft, Alfred-Nobel-Str. 50, D-40789 Monheim, Germany Bayer CropScience AG
* Corresponding author: Jessica.Bonham@bristol.ac.uk

1 Supplementary Information: Conductivity and Mobility

The conductivity of a solution, κ in S m^{-1}, depends on how many ions are present in the solution:

$$\kappa = \Lambda_m c, \tag{1}$$

where Λ_m is the molar conductivity of the solution and c is the concentration of the electrolyte[1].

Λ_m varies with concentration. One reason for this is that the ions do not always fully ionize in solution. For strong electrolytes, where substances are fully ionized, the conductivity depends only slightly on the molar concentration whereas for weak electrolytes, where substances are not fully ionized, the conductivity is highly dependent on the molar concentration[1]. To gauge the extent of charging in the microgel samples, in the calculations that follow, it is assumed that in non-aqueous solvents, all the microgel particles ionise with either a +1 or -1 charge, see Fig. 1.

Another reason that Λ_m depends on concentration is due to interactions between the particles. For strong electrolytes at low concentrations, the molar conductivity varies linearly with the square root of concentration, as described by Kohlrausch's law:

$$\Lambda_m = \Lambda_m^\circ - Kc^{1/2}, \tag{2}$$

where K is the correlation factor. Λ_m° is the limiting molar conductivity, defined as the molar conductivity in the limit of zero concentration and can be expressed as the sum of contributions from individual ions:

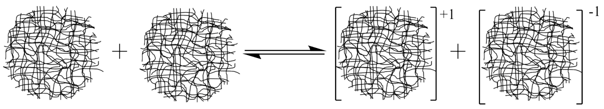

Figure 1: A cartoon of the possible charging mechanism of microgel solutions in non-aqueous solvents.

$$\Lambda_m^\circ = v_+\Lambda_+ + v_-\Lambda_-, \tag{3}$$

where Λ_+ and Λ_- are the limiting molar conductivities of the cations and the anions respectively and v_+ and v_- are the numbers of cations and anions per formula unit of electrolyte. The limiting molar conductivities of ions can be determined from the ionic mobilities (μ_i) using:

$$\Lambda = v\mu_i F, \tag{4}$$

where v is the valency of the ions. Faraday's constant is defined as $F = N_{\mathrm{av}}e$ where N_{av} is Avagadro's number and e is the elemental charge. The mobility of an ion is calculated using:

$$\mu_i = \frac{ve}{6\pi\eta r_{\mathrm{H}}}, \tag{5}$$

where η is the solvent viscosity and r_{H} is the particle hydrodynamic radius. Using Eq. 4 and Eq. 5 it is possible to link measurable and theoretical quantities and hence for a symmetric $v^+ : v^-$ electrolyte Eq. 3 becomes:

$$\Lambda_m^\circ = v(\mu_i^+ + \mu_i^-)F \tag{6}$$

The square root concentration dependence of the molar conductivity, seen in Eq. 2, can be explained in terms of the ionic atmosphere around each ion. When the central ion moves, the ionic atmosphere around it also moves, however it does not move immediately and thus the atmosphere becomes incomplete just in front of and behind the central ion. The centre of charge consequently becomes displaced and retards the moving ion, this is known as the relaxation effect. Additionally, the central ion experiences a viscous drag, which is enhanced in the presence of the ionic atmosphere because the ionic atmosphere moves in the opposite direction to the central ion. The enhanced viscous drag is know as the electrophoretic effect, which reduces the mobility and hence the conductivity of the ions[1].

The Debye Hückel Onsager theory attempts to quantify these complicated concentration effects and leads to the Kohlrausch-like expressions:[1]

$$K = A + B\Lambda_m^\circ, \tag{7}$$

with

$$A = \frac{v^2eF^2}{3\pi\eta}\left(\frac{2}{\epsilon RT}\right)^{1/2}, \tag{8}$$

$$B = \frac{bv^3eF}{24\pi\epsilon RT}\left(\frac{2}{\epsilon RT}\right), \tag{9}$$

where ϵ is the electric permittivity of the solvent, R is the gas constant, T is the temperature. b=0.586 for a 1:1 electrolyte. The agreement of the Debye Hückel Onsager theory with experimental conductivities is good at very low molar concentrations[1].

The Debye Hückel Onsager theory can be used to predict the conductivities of a suspension of charged microgel particles in solution using r_{H} and η and assuming a 50:50 split of $v = +1$ and $v = -1$.

References

[1] P. Atkins and J. de Paula, *Atkins' Physical Chemistry*, Oxford Univeristy Press, 7th edn., 2002.
